# Community-based anticipatory prescribing during COVID-19: a qualitative study

**DOI:** 10.1136/bmjspcare-2022-003597

**Published:** 2022-06-01

**Authors:** Bárbara Costa Pereira Antunes, Ben Bowers, Stephen Barclay, Joshua Gallagher, Riccardo Conci, Louisa Polak

**Affiliations:** 1 Department of Public Health and Primary Care, University of Cambridge, Cambridge, UK; 2 University of Cambridge School of Clinical Medicine, Cambridge, UK

**Keywords:** COVID-19, drug administration, home care, terminal care, clinical decisions

## Abstract

**Objectives:**

To understand healthcare professionals’ experiences of delivering anticipatory prescribing (AP) during the first wave of the UK COVID-19 pandemic.

**Methods:**

Semistructured qualitative interviews were conducted with a purposive sample of 16 healthcare professionals involved in community palliative care. Data were analysed inductively using thematic analysis.

**Results:**

Some of practitioners’ fears about the pandemic’s impact on delivering AP had not been realised during the first wave. Among patients with COVID-19 for whom community end-of-life care was deemed appropriate, deaths were perceived to be relatively easy to palliate with standard medications. These deaths were typically too rapid for AP to be appropriate or feasible. For non-COVID deaths, providing timely AP was more challenging: although community nurses and some palliative specialists continued to visit patients regularly, general practitioners did many fewer visits, moving abruptly to mainly remote consultations. This left some community nurses feeling undersupported, and prompted some palliative specialists to increase their direct involvement in AP. Several other changes were widely welcomed: collaboration to maintain drug supplies, adoption of online meetings and paperless practice, enhanced specialist helplines and a new policy allowing reuse of medication in care homes. The inclusion of more non-injectable options in AP protocols allowed clinicians to offer selected patients more choice, but few had yet done this in practice. No participants reported changing their prepandemic practice regarding administration of AP by lay caregivers.

**Conclusions:**

Accomplishing AP during a pandemic was challenging, requiring healthcare professionals to make rapid changes to their systems and practices. Some changes may produce lasting improvements.

**WHAT IS ALREADY KNOWN ON THIS TOPIC**

- The pandemic increased demand for community palliative care.
- Practitioners’ concerns included supplies and staff capacity to deliver anticipatory prescribing (AP).

**WHAT THIS STUDY ADDS**

- Beneficial changes in practice included reuse of medication in care homes, more paperless practice and improved collaboration.
- Remote consulting was controversial, affecting interprofessional role boundaries.

**HOW THIS STUDY MIGHT AFFECT RESEARCH, PRACTICE AND/OR POLICY**

- To make AP pandemic proof in future, practitioners and commissioners need to address ongoing workforce challenges and plan robust integrated systems.
- Patients and families’ experiences of AP warrant urgent research.

## Introduction

Pandemics produce an increase in the number of deaths in the community and often a rapid rise in demand for community palliative care.^1^ COVID-19 illustrates this: over the year from April 2020 to March 2021, the number of deaths at home rose by 41%, bringing implications for planning and organisation of palliative care.^2^ Two recent reviews^3 4^ identify a paucity of evidence to support such planning.

Anticipatory prescribing (AP) is the prescription of medication ahead of possible clinical need, for patients in their last weeks of life. This intervention is recommended practice in several countries and is widely considered by healthcare professionals to be key in ensuring rapid and effective symptom control for dying patients and in preventing admissions close to the end of life.^5 6^ Injectable formulations are often prescribed and are usually administered by visiting nurses, paramedics or doctors in the UK, Canada and Norway.^6^,^8^ This prescribing is just one component of the complex intervention of AP; other essential elements include the timely supply and administration of medication, and the clinical assessments and conversations that ensure its appropriateness.^8^,^11^

Early in the COVID-19 pandemic, healthcare professionals across the UK and Ireland described plans to modify their AP practice to address possible medication and staff shortages.^12^ National guidance was rapidly produced about managing community deaths from COVID-19,^13^ exploring alternatives to injectable medication^14^ and supporting relatives to administer palliative medication.^15^

Our study aims to examine the way healthcare professionals provided community palliative care during the first wave of the COVID-19 pandemic in the UK, with a particular focus on AP. By providing a detailed account of the way they addressed the challenges of delivering AP during this period, we seek to strengthen the evidence base needed to optimise future pandemic responses.

## Methods

This descriptive qualitative study follows an online survey about AP undertaken in April 2020, near the start of the UK COVID-19 pandemic^12^; respondents were healthcare professionals involved in palliative care in the UK and Ireland. To build on the overview provided by this survey, a subsequent qualitative study was undertaken to extend and deepen understanding of healthcare professionals’ experiences of delivering AP during the first wave of the pandemic.

### Reflexivity

The group of authors includes clinicians experienced in providing community palliative care (including AP) as well as those with an established track record of conducting and publishing qualitative research. Although this extensive experience is obviously a strength of the group, we are conscious that it inevitably shaped all components of the research, from participant selection and data collection through to analysis and the work of situating our findings in relation to the existing literature.

### Recruitment

118 survey respondents indicated willingness to consider taking part in a follow-up interview study. Of these, 31 were approached to take part and 16 agreed to be interviewed. Purposive sampling sought participants from a range of professional groups and geographical locations across the UK.

### Data collection

Semistructured interviews were conducted by Zoom and by phone between September and November 2020. Interviews lasted between 25 and 56 min, with two researchers (BCPA, JG) using a short topic guide (online supplemental appendix 1) which was continually adapted in response to insights from earlier interviews. All data were professionally transcribed verbatim, checked for accuracy and anonymised.

### Analysis

Data were interpreted inductively using constant comparison and thematic analysis^16^ by two researchers (BCPA and LP) who read all transcripts, coding them independently before discussing their findings; a third researcher (RC) read nine transcripts and discussed coding with BCPA. Though participants talked about many aspects of palliative care during the pandemic, for this paper our analysis focused on their experiences of AP.

## Results

Sixteen clinicians were interviewed. Table 1 describes their clinical roles and geographical locations.

**Table 1:** Participants’ clinical role and geographical location

Number of participants and clinical role	Region
3 Pharmacists	South EastEast of England
6 Palliative care consultants	South WalesLondonSouth EastYorkshire and the HumberNorth East
2 Palliative care nurses	Yorkshire and the HumberLondon
3 General practitioners (GPs)	East of EnglandSouth WestNorth East
2 Community nurses	East of EnglandSouth East

Two broad challenges were identified by participants involved in AP during the first wave of the COVID-19 pandemic in the UK. The first challenge concerned two medication issues: different palliative prescription needs for patients dying of COVID-19, and the need for alternative medications for all patients in case stocks ran out or administration was delayed. The second challenge concerned healthcare delivery: the pandemic disrupted many elements of the established systems that supported and enabled timely AP. We will first examine each of these challenges and the changes that were implemented to address them, and then present participants’ reflections on the future of these changes in AP practice.

### AP drugs and delivery routes

Many participants made a distinction between caring for people dying of COVID-19 and those dying from other causes.

#### Palliation for COVID-19

Several clinicians pointed out that the course of COVID-19 illness in community settings meant that end-of-life prescribing could seldom be ‘anticipatory’. One participant who worked in an area where AP had been very well embedded within community healthcare for years described setting up a different protocol for COVID-19 deaths:

If you tap in End-of-Life Care you get all the instructions for Anticipatory Prescribing at End of Life, and then there would be a link sort of for End-of-Life covid and you would link across, or you could tap in separately for covid. Because we felt it was, distinction was really important […] to make sure that people still thought of things in a slightly different way. (Participant 1: GP)

Rather than using their usual anticipatory medication prescription processes for these patients, they set up ‘covid treatment packs’ to address the need for an immediate response to acute distress.

For our [usual] Anticipatory Prescribing at End of Life, there we’re talking about […] the person who’s starting to deteriorate, is it going to be a good thing to get something into the house in the next 48 hours or so […] whereas the Covid drugs it was about immediacy. (Participant 1: GP)

The choice and dosage of anticipatory medications for people dying of COVID-19 in the community were mentioned by most participants, but not generally presented as problematic. This contrasts with survey data from the beginning of the pandemic,^12^ where respondents reported concerns about the likely need to modify their usual prescribing to palliate COVID-19 deaths outside hospital. In the autumn of 2020, with more experience of looking after this group of patients, interviewees described it as turning out to be less challenging than they had initially feared:

We thought we’d have really ill patients in community and care homes. There were hardly any in community and in care homes although lots of people died [….] in care homes […] we found that actually they died quite peacefully. They might need one or two injections of midazolam or morphine for breathlessness, many of them they didn’t need anything. So actually all those oh, ‘what ifs’ didn’t come to pass. (Participant 9: Palliative care consultant)

#### AP for non-COVID patients during the first wave of the pandemic

While these data indicate that ‘what ifs didn’t come to pass’ in relation to community COVID-19 deaths, participants did report producing alternatives to their existing prescribing protocols in response to two potential threats: drug shortages, and difficulty getting clinicians to administer injectable medication quickly. These concerns were very prominent in the April 2020 survey responses, but interviewees reported that the anticipated problems had not subsequently materialised in many places. Nonetheless, many participants expressed satisfaction at having set up guidelines in case of future disruption: ‘it’s all in place if it lifts off again’ (Participant 1: GP).

Several participants described writing protocols about alternative injectable medications to those used routinely, providing guidance on equivalent dosages. Additionally, most protocols included some alternatives to injectables for people no longer able to take oral medication: maximising use of transdermal opioid patches where rapid dosage escalation was not required, and using buccal, sublingual or rectal routes for rapid palliation. These non-injectable options were designed to be usable by the patient’s family or other lay carers. Many involved off-licence use of preparations licensed for oral use:

What we did was write a guideline initially and then pull together lots of different guidelines from the UK into one big one, that looked at […] alternative routes to get something into to somebody’s body. […] things like MST tablets rectally, Oramorph liquids sublingually. (Participant 2: Pharmacist)

In practice, although some clinicians reported using opioid patches more than before, none had needed to switch to the non-standard injectable drugs in the new formularies, and none reported resorting to non-injectable alternatives in any new or unusual way. Most participants said that, in their areas, drug supplies had been maintained and community nursing services had been able to continue visiting to administer injections or set up syringe drivers:

[Interviewer: ‘And how about sublingual, buccal route administration?’]

I was aware that that was in the guidance but […] I didn’t need to use it at all. […] our district nurses were still going in and we have an out-of-hours palliative care team, I don’t think there was any overwhelming of services in [place name] during the first wave. (Participant 3: GP)

Thus, the medications participants reported prescribing for non-COVID-19 palliation during the first pandemic wave were not significantly different from those used in ‘normal’ circumstances.

### Accomplishing AP in a safe and timely way

Participants described notable changes to the practical aspects of AP, identifying disruption at every stage of the process. Most spoke far more about service delivery than about medication choices, a reminder that the drugs themselves constitute just one element of a complex intervention. AP involves several intermingled elements, all of which were complicated by the pandemic: communicating within the healthcare team and between clinicians and patients (and their families and carers); recording, prescribing and authorisation; providing medication; and administering medication to patients. We consider changes to each of these elements in turn, before looking at the way concerns about safety and risk shaped these changes.

#### Talking with patients and colleagues

A dominant theme was the sudden reduction in face-to-face interactions and the corresponding marked increase in the use of phone and video to enable communication. Participants gave widely differing accounts of this change, with some of the differences related to the three different contexts in which remote communication was used: for meetings about AP guidelines; for discussing a patient’s care; and for clinical consultations.

- *Remote guideline meetings*

Participants who worked with colleagues to produce local or national AP guidance almost unanimously reported that meeting remotely had been beneficial; it had made organising meetings quicker and easier, increased participation and facilitated collaboration with colleagues across a wider geographical area:

We work a lot faster because we do things remotely. So you’re no longer waiting to get everybody in the same building, into the same room for a meeting. (Participant 2: Pharmacist)

- *Remote discussions about individual patients*

Most participants reported that meeting colleagues remotely to discuss patient care was satisfactory and had time-saving benefits:

The GP surgeries have continued with their Gold Standards meetings but we’ve done them either by Zoom or Microsoft Teams […] so hopefully we’re discussing those cohort of patients and make sure things are in place at the appropriate time. (Participant 5: Palliative care nurse)

However, not everyone agreed. A few participants indicated that, for them, the loss of face-to-face interaction did matter in relation to talking with general practitioners (GPs) about individual patients:

We still have good relationships with the doctors and we share the same computer system 99% of the time, and you know, they’re at the end of a phone to discuss issues, but it’s not quite the same as doing a face-to-face chat with them. (Participant 14: Community nurse)

- *Remote consultations with or about patients*

The perception that a remote conversation is ‘not quite the same’ as face to face was more prominent in relation to conversations with patients and their families. This topic divided opinion, the division largely following an interprofessional boundary between doctors and nurses, with the nurses reporting dissatisfaction with GPs’ marked restriction of face-to-face encounters with patients. Their dissatisfaction focused both on the perceived disadvantage to patients and on the effect on the nurses themselves; some spoke of feeling poorly supported, while others indicated resentment at being expected to carry on as usual while GPs stayed safely remote.

I think GPs could be a bit more onboard, I mean they’re all saying ‘oh yeah, we’re doing whatever’ but […] certainly in community nursing I don’t really know what they’re doing because they don’t seem to really visit patients or go out or they might have a call-car or something that goes round but […] I don’t think it’s that satisfactory really. (Participant 11: Community nurse)

On the other hand, GPs and palliative care doctors described continuing to work hard, presenting their shift to predominantly remote consulting as necessary for infection control. While some did mention that it is challenging to consult remotely with patients approaching the end of their life, most said it was manageable, particularly where they had some pre-existing relationship with the patient and their family or carers. In relation to working with carers in a care home, a GP commented:

I used to go in three times a week for probably the best part of four years so having me on the phone I have that pre-existing relationship with them so they’re happy to take that advice. They don’t need to see me face-to-face to be happy with my advice to them. (Participant 3: GP)

The same participant later implied that, unlike talking with carers, they had expected remote consulting with relatives to be unsatisfactory, but they felt this had turned out not to be the case:

The fact that the relatives dealt with the remote stuff quite so well, that really surprised me […] there’s things I would never have done remotely that actually they’re not that different. (Participant 3: GP)

So, having tried remote consulting close to the end of life, some doctors found it surprisingly satisfactory. The only doctor who spoke strongly against this said they had not tried it and stated that they felt patients really appreciated meeting clinicians face to face:

Just the fact you’ve come through the door […] and haven’t tried to diagnose them over a video, is just so valued. (Participant 10: Palliative care consultant)

These few participants’ negative views predated the widespread demand in 2021 for a return to more face-to-face consultations in the UK. These participants were also outliers in terms of the unhappiness they expressed about other aspects of their work during the pandemic.

One form of remote interaction that all participants spoke highly of was telephone helplines, provided by community palliative care specialists for patients, families and clinicians. Most areas already had specialist helplines, but many extended them during the COVID-19 pandemic:

We at that time did not have a local consultant 24-7 advice service. It was provided by a hospice in [city] and so we set up a 24-7 consultant advice service so that people had access, you know, to clinicians they were more likely to know and more likely to feel able to phone. (Participant 9: Palliative care consultant)

To function safely, clinicians staffing helplines need access to the patient’s records. One participant highlighted the value of the Coordinate My Care (CMC) electronic record:

A patient they panic, they call 999 and the ambulance, they do check the CMC, and because now that we have got a Your Lifeline Service 24/7, […] they will ring them directly or ring our office directly, and we actually able to speak to the ambulance crew and identify what’s the situation. (Participant 6: Palliative care nurse)

#### Records, prescriptions and authorisation forms

Widespread changes to AP ‘paperwork’, including adopting electronic prescriptions, were welcomed by participants. The prepandemic starting point for these changes varied between geographical regions, ranging from fully electronic to mainly paper systems. Participants were almost unanimous about the benefits of moving away from paper, citing speed, efficiency, clarity and shareability between clinicians in different places. The accelerated adoption of electronic AP paperwork was widely seen as a positive benefit of the pandemic:

The positive thing from Covid is actually speed up people using a bit more electronic version of things […AP has] speeded up with the pan-London MAAR [Medicine Authorisation and Administration Record] charts […which] also minimise the time to for the nurses to go to and fro to get the GP to sign up. (Participant 6: Palliative care nurse)

Clarity is particularly important for safe AP where several clinicians may be updating the patient’s clinical record about findings, preferences and decisions taken, and amending prescriptions. One GP talked about hoping to move away soon from a paper Kardex, the medication authorisation form often used in care homes:

An electronic form of that green Kardex would be good [….] then I can adjust it remotely as well if the person needs an increased dose or whatever I can do that without there being this physical transfer of a bit of paper. (Participant 3: GP)

Shareability, another advantage of paperless systems, is essential for safe AP. It has long been an aspiration in the National Health Service to establish a comprehensive electronic patient record, including end-of-life care preferences and plans, to be used by all clinicians in the community. Where this existed or was achieved during the first wave of the pandemic, it was widely welcomed by participants. The following excerpt also alludes to the more challenging aspiration to enable clinicians to access records across the primary-secondary care divide:

Access to the shared record makes a massive difference and I think… well, for a number of years now we’ve been able to access the hospital record as well. (Participant 5: Palliative care nurse)

#### Getting medications to the patient: dispensing and delivering

Potential supply problems affecting AP were a concern for most participants. Several said the national shortages of commonly used drugs that were widely anticipated at the start of the pandemic did not materialise during its first wave. A few participants did report local shortages, while many others described anxiety that these might have arisen. There was potential for a vicious circle through which fear of shortages exacerbated actual shortages:

Everybody got out there and tried to be proactive and planned ahead as much as they could, and went round anticipatory prescribing left, right and centre really for anyone that might need it within the next few months, which I can see completely why that would seem like a good idea but what actually happened was that ran all the stocks out. (Participant 4: Palliative care consultant)

This participant went on to explain that the problem was solved in their region by coordination and collaboration to create a robust system: an agreed list of drugs was stocked by designated palliative care pharmacies in the community and out-of-hours GP services, with additional funding to pay for unused expired stocks. Further additional funding enabled the local hospital to offer back-up from their pharmacy, with an on-call pharmacist service for the community. Clinicians then felt confident enough to stop ‘panic buying’, instead prescribing closer to an expected death:

We stopped anticipatory prescribing unless we thought it was a few days because we had confidence in the emergency measures. (Participant 4: Palliative care consultant)

Getting medications to the patient’s home was also complicated by pandemic restrictions, particularly for people advised to shield. Some pharmacies expanded their delivery service; one region also commissioned a new emergency delivery service for palliative care, using couriers to deliver AP medications to a patient’s home within 2 hours.

One change that was unanimously welcomed was the new standard operating procedure (SOP)^17^ temporarily altering guidance about medication management in English care homes and hospices during the pandemic. Previously, when a resident died their medication had to be disposed of; the new SOP allows care homes to use these drugs for another resident when urgently needed, following the issue of a new prescription. Participants described three advantages of this reuse: it reduced the work of arranging for medications to be dispensed and delivered, the delay before administering medication and the wastage of supplies at a time of potential shortages.

#### Administering medication to the patient

Administering medication was another aspect of AP where participants anticipated changing their practice during the pandemic’s first wave but turned out not to need to do so: community nursing teams were able to continue visiting patients to give injectable medication in a timely way. Additionally, some described establishing new ways of working with paramedics, who are already allowed to carry and administer opiates:

We’ve done lots of work with the paramedics […] they can’t give the drugs unless there’s what we’ve called a shared decision-making procedure where they need to get a healthcare professional on the phone, ideally one that knows the patient […] so they’re not taking on all of the decision-making themselves. (Participant 4: Palliative care consultant)

Participants had a range of views about inviting family and carers to administer medication, by injectable, buccal and rectal routes. Although oral and transdermal routes are not generally considered in the context of AP, participants described using them for patients dying with COVID-19 in the community, sometimes supporting relatives to administer them. A few areas had already established protocols for assessing and training relatives to administer injectable medications. Some who had not yet set this up pointed out the desirability of offering the patient choices regarding the route of administration, and the option for relatives to give medication. Others expressed significant reservations, both about the feasibility of training people remotely and about potentially burdening relatives with considerable responsibility:

We did have quite a lot of discussion about if you’d given an injectable and somebody died very quickly afterwards, as a family member would you think you’d killed that person, […] you have to be very selective and it wouldn’t be right for everybody. (Participant 1: GP)

A key concern was the need for adequate support for anyone administering medication close to the end of life, be they lay carers or community nurses:

[Interviewer: ‘What would be your number one worry concerning anticipatory prescribing at the moment?’]

Um…having a really complex patient that I hadn’t faced before and not having the specialist support there at that time. (Participant 14: Community nurse)

#### Risk, safety and governance

Safety was mentioned by several participants who cited possible diversion or excessive use of drugs left in the home. However, others felt that these risks were overstated:

If you limit the amount of drugs that are put into somebody’s home, our Just in Case is three doses, it’s very limited what harm you can do with three doses, and if they suddenly start ordering lots more well you know something’s wrong don’t you. So it’s not an open-ended prescription that you can just pour drugs into people. (Participant 1: GP)

A few participants emphasised the tension between the risks of misuse and the risk of undertreating a distressed patient:

It’s a risk reduction […] a balanced judgement between the risk of the medicines not being there and the risk of there being too many medicines around. (Participant 9: Palliative care consultant)

Both these concerns are present in relation to AP in ‘normal’ times, but several participants indicated that the pandemic added weight to ‘the risk of the medicines not being there’, because of the extra difficulty of timely delivery. Consequently, the pandemic had made some less risk averse:

I think that we’ve been able probably through Covid to start to get a more proportionate analysis of risk and become, rather than risk averse to become risk aware so you’re recognising what the risks might be [… and] can justify the actions you take. (Participant 12: Palliative care consultant)

Regulation was a topic with a clear difference between professional groups. Several doctors felt regulation was unnecessarily burdensome, while some pharmacists spoke of the great caution needed to make changes:

We need a bit more time to […] just kind of make sure that what we’re doing is right and proper. Because what you can do when you’re in a level four pandemic is very different from what you then do when you’re not in that situation. (Participant 2: Pharmacist)

This contrasts with the tone of some doctors’ accounts:

So we’ve pushed through protocols that would normally have got stuck in some kind of ‘you can’t do that’ quagmire. (Participant 13: Palliative care consultant)

### Looking ahead: which changes should be kept?

Participants were asked about changes to AP that they considered to be positive and worth keeping after the pandemic. These have largely been covered above: paperless practice was unanimously popular, as were electronic systems to coordinate medication supplies, reuse of medications within care homes and remote interprofessional meetings with the associated increased collaboration across geographical boundaries. Remote communication with and about patients divided views sharply, and there were some interprofessional differences regarding the regulations governing prescribing and dispensing. Inviting relatives to administer AP medication had not changed significantly, but guidance had been produced and was perceived as being possibly useful in the future.

## Discussion

Our study shows that although medication choices, dosage and administration were affected less than expected, the pandemic disrupted most other aspects of the AP process. This stimulated rapid changes, some of which were perceived as beneficial by many participants. By examining changes in AP practice during the first wave of the pandemic, the study contributes to two growing bodies of knowledge: one concerns pandemic-related changes in community healthcare, and the other concerns AP.

Regarding pandemic-related changes, our results and conclusions complement those of a large survey^18 19^ of primary care providers’ experiences of palliative care during the UK pandemic’s first wave. Like those survey respondents, our participants emphasised the importance of interprofessional collaboration and support. The pandemic was seen as accelerating several welcome changes; these included increased use of technology, although there were some concerns about remote consultations. In a study of the pandemic experiences of staff in care homes,^20^ with a focus on caring for residents with COVID-19, staff spoke of the importance of AP, describing the rapid trajectory of COVID-19 deaths in their patient group and the usefulness of having medication available for buccal administration where a healthcare professional could not attend immediately; these findings mirror those of our study.

Some of our findings relate to changes that are not specific to AP. For instance, recent literature has examined the central role played by community nurses in responding to COVID-19,^19 21 22^ something our study also highlights. The rapid shift to remote consulting has been extensively discussed and studied^23^; we identified interprofessional differences regarding the advantages and disadvantages of this shift, as did the primary care survey cited above,^18 19^ which found differences were associated with tensions between the professional groups. We would agree with these authors about the need to rebuild trusting relationships between the different groups who provide community palliative care.

Contributing to the knowledge base that informs AP, our findings are of particular relevance where they identify pandemic-driven changes that might become permanent.^12^ Rather than the specifics of medication choices and doses, these changes concern the practical and organisational aspects of AP, which is a complex intervention whose components interact.^5 7 9 11^ Our data illustrate these interactions. For example, robust systems to provide urgent supplies of medication affected prescribing practice: by enabling clinicians to prescribe later and less, these supply systems made shortages less likely and reduced wastage, and averting shortages meant that guidelines about using alternative, non-injection routes to deliver medication were not needed. Some of our participants welcomed the renewed interest in these alternatives as a way of giving patients a choice of delivery route where appropriate, thus enabling shared decision-making, a process endorsed as essential within international guidance on palliative care.^24^

Offering patients a choice about drug delivery route seems uncontentious, but several participants expressed reservations when asked about the related topic of offering to train relatives to administer medications close to the end of life. These reservations echoed those identified in some prepandemic studies,^6 25 26^ although others have been more positive.^27^,^29^ The pandemic increased the difficulty of ensuring that relatives are comfortable taking on the task of giving medication: those participants who were willing to consider training and supporting relatives pointed out that doing this would be far more challenging remotely than face to face. While participants’ practice had not changed regarding asking relatives to give medication, there was considerable variation in their opinions between geographical areas, and also between giving non-injectables and injectable doses. National guidance for supporting friends and relatives to give injectable medications has been produced in Wales, based on a prepandemic feasibility trial^15^; it is a practice supported by English^30^ guidance, subject to the willingness and appropriate training of those involved. It is already usual practice in several regions of Australia for families to administer injectable medications.^6 27 28^ Drawing on the results of studies to date, a recent commentary^31^ emphasised that administering injectable medications is ‘a big ask’ for relatives and that those who decide to take it on need tailored training and ongoing professional support.

Our study has several strengths and limitations. Caution is needed in interpreting results of research conducted during a pandemic, which inevitably produces a time-specific snapshot. Relating the findings of our current study to the results of the survey 6 months earlier^12^ helps add some perspective, although the differing methods preclude direct comparisons. While it was useful to compare different professional groups in the analysis, self-selection and the small number in each group are further reasons for caution. The inclusion of a diverse range of participants, working in different regions, supports the transferability of the findings across the UK and to countries who have adopted similar healthcare service innovations during the pandemic. Focusing on one aspect of palliative care, AP, facilitated a detailed analysis that enabled us to draw some usefully specific conclusions. Key decision points in the analysis were debated by three of the researchers (LP, BCPA and RC), and final themes were refined and agreed by all the authors, helping to achieve a comprehensive and reflexive analysis.

Looking to the future, pandemics are known to create increased demand for community palliative care. Optimising responses to this demand requires a detailed understanding of palliative care practices during a pandemic. To develop this understanding further, it would be valuable to mirror our study by exploring patients and families’ experiences of pandemic palliative care, including AP. Additionally, re-examining healthcare professionals’ perceptions after the pandemic would help establish which changes have permanently shaped AP and other aspects of community palliative care. Our findings suggest that beneficial innovations in AP introduced during the pandemic could usefully be incorporated into postpandemic practice, although they would need tailoring to healthcare delivery and governance contexts in different countries and their effects will need to be carefully evaluated. Community palliative care practitioners need to reflect with their patients on the professional roles that are needed in the future, and work with colleagues to develop and integrate these, building in the capacity to deal with a potential future pandemic.

## Conclusion

During the UK COVID-19 pandemic’s first wave, healthcare professionals addressed significant challenges to meet the AP needs of an increased number of people dying in the community. This required them to attend to their prescription choices and to ‘stuff, staff and systems’.^1^ Understanding their experiences should help identify important questions for future research, and to shape the ongoing evolution of AP practice in the UK and internationally.

## supplementary material

10.1136/bmjspcare-2022-003597 online supplemental file 1

## Data Availability

Data are available upon reasonable request.
