# Supplementary material for: Community-based anticipatory prescribing during COVID-19: a qualitative study
Source: BMJ Support Palliat Care. 2022 Jun 1;14(e3):e003597. doi: 10.1136/bmjspcare-2022-003597 (PMC11672004; doi:10.1136/bmjspcare-2022-003597)
Supplement: online supplemental file 1 [file bmjspcare-14-e3-s001.pdf]

Appendix: topic guide for qualitative interviews

1. OPENING

Aims: to discuss:
- Changes occurred your area / more widely
- Changes persisted / returned to previous
- Expectation for future of AP practice

Estimated 30-60 minutes

PRESS RECORD
Verbal consent recorded
Could you start by saying what your role is and how are you involved in AP, what bits of the process do you do

2. MAIN QUESTIONS

a) Can we start by discussing the impact of the COVID-19 pandemic on your personal PEOLC practice and practice in your local area?

b) What was the impact of the pandemic on AP in your personal practice and your local area?

a. *Prescribing*
b. *Route of administration, esp. sublingual / buccal route*
c. *Person administering AP drugs, esp. family / informal carers or social carers*
d. *Use of phone / video to support AP*
e. *Use of syringe drivers*

1 What are your views of changes that took place?

2 To what extent are those changes still in place or has practice returned to how it was pre-pandemic?

3 Looking to the future, how do you see AP developing and changing?

4 What is your number 1 worry concerning AP at the moment, when we don't know what will happen with COVID?

5 regarding support for healthcare professionals, I mean in terms of their wellbeing, not so much clinical decision making, do you have anything in place?

C. CLOSING

Anything else would like to add?

Thanks
